# Proliferative glomerulonephritis with monoclonal IgG deposits; an unusual cause of de novo disease in kidney allograft

**DOI:** 10.15171/jnp.2017.36

**Published:** 2017-04-05

**Authors:** Sabiha M Hussain, Kalathil K Sureshkumar

**Affiliations:** Division of Nephrology and Hypertension, Department of Medicine, Allegheny General Hospital, Pittsburgh, USA

**Keywords:** De novo disease, Kidney allograft, Immunoglobulin deposition

## Abstract

**Background:**

Proliferative glomerulonephritis with monoclonal IgG deposits (PGNMID) is a newly described and rare entity that can develop in native and very rarely in transplanted kidneys. We present a patient who developed de novo PGNMID in the kidney allograft along with a review of the literature.

**Case Presentation:**

A 38-year old female with type 1 diabetes who underwent successful simultaneous pancreas-kidney (SPK) transplantation 6 years earlier presented with rising serum creatinine, nephrotic range proteinuria and microhematuria. She underwent extensive work up and kidney allograft biopsy revealed mesangial expansion and hypercelluarity on light microscopy, mesangial staining for IgG3, kappa light chains, C1q and C3 on immunofluorescence and abundant mesangial electron dense deposits without substructures on electron microscopy. Serum and urine immunofixation electrophoresis were negative. A diagnosis of de novo PGNMID was made. Patient’s proteinuria improved and serum creatinine stabilized with conservative therapy.

**Conclusions:**

PGNMID can rarely develop in kidney allograft as recurrent or de novo disease and may be mislabeled as transplant glomerulopathy if careful immunofluorescence and electron microscopy are not performed on biopsy specimens. Further studies are needed to better understand the pathogenesis of this disease entity and to develop optimal therapeutic approaches.

**Implication for health policy/practice/research/medical education:**

Proliferative glomerulonephritis with monoclonal IgG deposits (PGNMID) can develop in kidney allografts as a recurrent disease and very rarely as de novo. A high index of suspicion is needed to make the diagnosis. PGNMID can be mistakenly labeled as transplant glomerulopathy if immunofluorescence and electron microscopy are not performed on the allograft biopsy with light microscopy showing changes of membrano-proliferative glomerulonephritis (MPGN). Further studies are needed to better understand the pathogenesis of this disease entity and to develop optimal therapeutic strategies.

## 1. Introduction

Proliferative Glomerulonephritis with monoclonal IgG deposits (PGNMID) is a newly described entity characterized by monoclonal IgG deposits in the kidney. The incidence of PGNMID in the native kidneys is about 0.17% (1). It can mimic other monoclonal diseases such as light and heavy chain deposition disease, light and heavy chain amyloid, immunotactoid and fibrillary glomerulonephritides and type I cryoglobulinemic glomerulonephritis. In PGNMID, monoclonal deposits are strictly glomerular consisting of single light chain isotype and single heavy chain subtype, most commonly IgG3 k (2). Membranoproliferative or endocapillary patterns of glomerulonephritis are seen on light microscopy with glomerular electron dense deposits on electron microscopy (1,3).

PGNMID can develop in native kidneys and occasionally in kidney allograft, where it presents mostly as recurrent disease within two years after transplantation or very rarely as de novo manifesting years later. In the kidney transplant, PGNMID can present as nephrotic range proteinuria with or without hematuria and allograft dysfunction (4). We present a patient who developed PGNMID 6 years after simultaneous pancreas-kidney (SPK) transplantation.

## 2. Case Presentation

**Figure 1:**
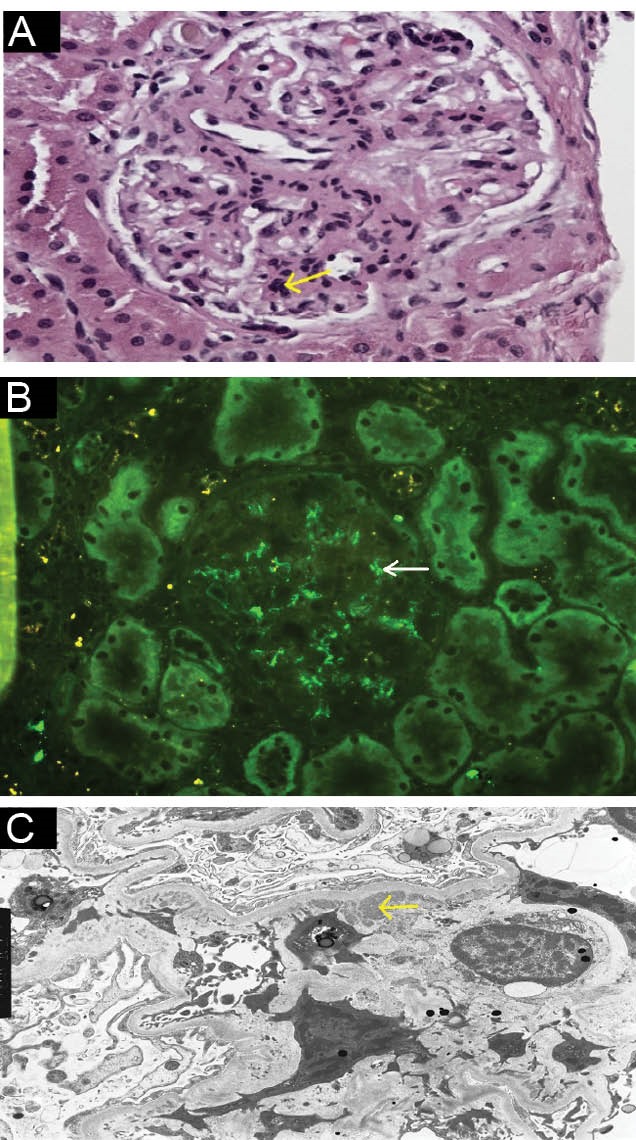
(A) Light microscopy showing increased mesangial matrix and cellularity (Hematoxylin-Eosin x400). (B) Immunofluorescence microscopy showing 2+ granular mesangial IgG3 staining. (C) Electron microscopy showing mesangial electron dense deposits (x3900).

A 38-year old white female with type 1 diabetes mellitus complicated by retinopathy, gastroparesis and end-stage renal disease (ESRD) underwent deceased donor SPK transplantation in November of 2007. Cytomegalovirus (CMV) serology was negative in both donor and recipient. Patient was not sensitized and received perioperative induction therapy with rabbit-antithymocyte globulin followed by maintenance immunosuppression with tacrolimus (dose adjusted for targeted trough level of 8-10 ng/mL during first year, 6-8 ng/mL during second year and 4-7 ng/mL subsequently) and mycophenolate mofetil (500 mg twice daily) with early steroid withdrawal. Trimethoprim/sulfamethoxazole (400/80 mg) once daily was used for prophylaxis against Pneumocystis jiroveci infection. Allografts started functioning soon after transplantation without delayed graft function. Serum creatinine stabilized at 1.3 mg/dL and patient remained euglycemic. Six years later, serum creatinine increased to 1.8 mg/d with a blood urea nitrogen level of 20 mg/dL. Urinalysis showed evidence for proteinuria and microhematuria. Other pertinent laboratory values were as follows: WBC 3.29 µ/mL, hemoglobin 10.7 g/dL, platelets 209 µ/mL, serum albumin 3.7 g/dL, serum total cholesterol 167 mg/dL, fasting blood glucose 102 mg/dL, and trough tacrolimus level 5.4 ng/mL, along with negative CMV, BK virus and Epstein-Barr virus PCR. She had proteinuria of 5 g/d. Patient underwent extensive work-up. Serum ANA, anti-double-stranded DNA, cryoglobulins, rheumatoid factor, anti-streptolysin O, proteinase-3 and myeloperoxidase ANCA, as well as hepatitis B and C antibodies were negative. Levels of serum complements and immunoglobulins were normal and no monoclonal bands were observed on serum and urine immunofixation electrophoresis. Serum free light chain assay revealed elevated free kappa (33.8 mg/dL, normal range 3.3-19.4) and normal lambda (25.1 mg/dL, normal range 5.7-26.3) fractions. Patient subsequently underwent kidney allograft biopsy. Light microscopy showed increased mesangial matrix and hypercellularity. Immunofluorescence revealed mesangial 2+ staining for IgG with specificity for IgG3 subclass, C3, C1q, IgM and 1+ staining for kappa light chains with negative staining for lambda light chain, IgA and subclasses of IgG1, IgG2 and IgG4 as well as peritubular capillary C4d. On electron microscopy, abundant mesangial granular electron dense deposits without substructures were seen. These findings were consistent with a diagnosis of PGMNID (Figure 1).

We opted for conservative treatment in our patient. She was initiated on lisinopril which she tolerated well. She remained normotensive. Her serum creatinine remains at 1.8 mg/dL with <100 mg/d proteinuria 14 months later.

## 3. Discussion

PGNMID can develop in kidney allograft, usually as recurrent disease whereas de novo disease is extremely rare (4-9). Recurrence tends to occur early, usually within first 2 years after transplantation, while de novo disease develops several years later (4,5). We present a patient with de novo PGNMID that developed in the kidney allograft 6 years following a successful SPK transplantation. To our best knowledge, there have only been two other cases of de novo PGNMID in kidney allografts described in the literature so far (5). Even though our patient did not undergo native kidney biopsy, it is highly likely that diabetic nephropathy caused ESRD based on clinical presentation and natural course of the disease.

Reported cases of PGMNID in kidney allografts are summarized in Table 1. Two case series each with four patients were reported by Nasr et al and Albawardi et al (4,5). PGNMID presents with varying degrees of proteinuria sometimes in the nephrotic range, with or without hematuria and allograft dysfunction. The disease generally occurs in adults, usually over 50 years of age, and is more common in white females. Natural course of the disease in the allograft is assumed to be similar to native kidney disease. Largest series on native disease was published by Nasr et al (1). Only 30% of patients in this study were found to have monoclonal spike in serum or urine and these bands were identical to heavy and light chain isotypes identified in the glomerular deposits. Serum complements were depressed in 10% of the patients. During a median follow up of 30 months involving 32 patients, 38% had complete or partial recovery, 38% had persistent renal dysfunction and 22% progressed to ESRD. Most of the patients had no detectable M protein even after long follow-up, and the authors suggested that PGNMID is likely not a precursor of myeloma (1). A recent study published by Batal et al identified eight cases of PGNMID after analyzing 659 real allograft biopsies (10). The authors did not mention whether any of these cases were de novo. The same study also reported a different set of 38 native kidney biopsy specimens with PGMNID. Interestingly 18% of these patients with native kidney involvement had evidence of hematologic malignancies mostly in the form of low-grade lymphomas (10). PGNMID in the native kidneys associated with chronic lymphocytic lymphoma was reported in two patients by Barbour et al (11).

**Table 1:** Reported cases of kidney allograft PGNMID in the literature

**References**	**Nasr et al** ^4^	**Albawardi et al** ^5^	**Sumida et al** ^6^	**Ranghino et al** ^7^	**Wu et al** ^8^	**Batal et al** ^9^
No. of cases	4	4	1	1	1	9
Recurrent/ de novo	All recurrent	2 recurrent, 2 de novo	Recurrent	Recurrent	Recurrent	No information
Native kidney disease	2 with MPGN and 2 with endocapillary proliferative patterns of PGNMID	2 with PGNMID, 1 with PKD, 1 with diabetic nephropathy	Endocapillary proliferative pattern of PGNMID	Crescentic endocapillary proliferative pattern of PGNMID	Mesangial proliferative pattern of MPGN	Unclear how many had biopsy. 2 with MPGN
Clinical presentation post-transplant	Proteinuria, microhematuria, rising creatinine	Proteinuria, rising creatinine	Proteinuria, microhematuria, rising creatinine	Proteinuria, rising creatinine	Proteinuria	1 with proteinuria, hematuria, rising creatinine. No data on the rest
Time from transplant to diagnosis	3-5 months	Recurrent: 12-13 months, De novo: 14-30 months	4 months	18 months	19 months	5 months to 22 years
Allograft biopsy findings	2 with mesangial proliferative and 2 with endocapillary proliferative pattern of PGNMID with monotypic glomerular IgG3 in all	2 with MPGN (glomerular IgG3 in one and IgG1 in other); 1 with BK nephropathy and mesangial IgG3;1 with ATN and mesangial IgG3	Endocapillary proliferative pattern of PGNMID with glomerular capillary and mesangial IgG2 staining	Endocapillary proliferative pattern of PGNMID with light chain kappa staining	Mesangial proliferative pattern of PGNMID with Glomerular IgG3 staining	7 with MPGN, 1 with ATN 6 had IgG stain (3 IgG3, 2 IgG 1). 2 had IgA stain
Treatment	Steroid + rituximab in 3 patients; steroid + cyclophosphamide in 1 patient	No specific therapy	High dose steroid	Pulse steroid + plasmapheresis + IVIG	Steroid +cyclo- phosphamide + double filtration plasmapheresis	Not mentioned
Outcome	Improved allograft function in 3 patients	Death with functioning graft in 2 (I cardiac, 1 infection); dialysis in 1; rising creatinine in 1	Improvement in allograft function and proteinuria	Initial improvement. Patient underwent transplant nephrectomy 6 months later	Infection related death with functioning graft	3 allografts failed, 3 functioning, no information available on 3 patients.

Abbreviations: ATN, acute tubular necrosis; MPGN, membrano-proliferative glomerulonephritis; PGNMID, proliferative glomerulonephritis with monoclonal immunoglobulin deposits; PKD, polycystic kidney disease.

Pathogenesis of PGNMID remains elusive. Recurrence in renal allograft suggests the possibility of a persistent circulating factor in the recipient. Glomerular deposition of a circulating non-deleted monoclonal IgG molecule followed by complement fixation and activation of downstream inflammatory mediators was suggested by some (2). Glomerular injury is thought to arise as a response to either intrinsic or extrinsic antigen that stimulates proliferation of one or more B-cell clones resulting in the production of monoclonal IgG, usually of IgG3 subtype which is quickly absorbed by glomeruli thus escaping detection by immunofixation. IgG3 subtype which is only 8% of total IgG has the highest molecular weight; greatest complement-fixing ability and is positively charged, all features rendering it intrinsically nephritogenic with high affinity for negatively charged glomerular constituents (4).

Histologic characteristics of PGNMID in kidney allograft biopsy are not different from those in native kidney biopsy (4,5). Light microscopy shows mesangioproliferative or endocapillary glomerulonephritis. Electron microscopy shows prominent granular mesangial and subendothelial electron dense deposits. Immunofluorescence clinches the diagnosis by showing staining for monoclonal IgG with one of the subtypes, most commonly IgG3 and either kappa (most common) or lambda subtype exclusively in the glomeruli. C3 and C1q staining can be positive indicating the activation of complement system but IgA and IgM stains are usually negative.

PGNMID needs to be differentiated from post infectious glomerulonephritis, primary membrano-proliferative glomerulonephritis (MPGN), transplant glomerulopathy (TG) and other monoclonal dysproteinemias with renal involvement such as type I cryoglobulinemic glomerulonephritis as well as immunotactoid and fibrillary glomerulonephritides. It differs from type I cryoglobulinemic glomerulonephritis by the absence of serologic evidence for cryoglobulinemia and lack of annular-tubular or fibrillar substructure by electron microscopy of kidney biopsy. Immunotactoid glomerulonephritis is associated with characteristic finding of microtubules of 30 to 50 nm in diameter on electron microscopy whereas fibrillary glomerulonephritis demonstrates the presence of Congo red negative randomly oriented fibrils measuring 16 to 24 nm. In the absence of immunofluorescence and electron microscopies, findings of PGNMID can be mistaken for TG, but IgG staining is usually weak in TG and lacks monoclonality (5).

There is no proven therapy for PGNMID and treatments attempted include high dose steroids, rituximab, cyclophosphamide and plasma exchange (4,6-8). In a handful of patients with recurrent disease in the allograft, response to rituximab and cyclophosphamide was observed which the authors attribute to early diagnosis of the disease with protocol biopsies (4). An extensive evaluation for underlying dysproteinemia which includes serum and urine electrophoresis with immunofixation, serum quantitative free light chain measurement and sometimes bone marrow biopsy is warranted. We opted for conservative management in our patient with lisinopril. Her serum creatinine remains at 1.8 mg/dL with <100 mg/d proteinuria 18 months later.

## 4. Conclusions

In summary, we present a case of de novo PGNMID that developed in the renal allograft 6 years after a successful SPK transplant along with review of literature on both recurrent and de novo PGNMID in renal allografts. The possibility of PGNMID should be considered in the differential diagnosis when light microscopy shows features of MPGN in renal allograft biopsies. Further studies are needed to better understand the pathogenesis of this disease entity and to develop optimal therapeutic approaches. Based on the evidence thus far, patients with a diagnosis of PGNMID should be monitored long term for the possibility of developing hematological malignancies.

## Acknowledgements

Presented in part as a poster at the National Kidney Foundation Spring Clinical Meeting, May 2015, Dallas, TX.

## Authors’ contribution

Authors contributed to the manuscript equally.

## Conflicts of interest

The authors declare that they have no conflicting interest.

## Funding/Support

None.
